# Monitoring the Methyl Eugenol Response and Non-Responsiveness Mechanisms in Oriental Fruit Fly *Bactrocera dorsalis* in China

**DOI:** 10.3390/insects13111004

**Published:** 2022-11-01

**Authors:** Yinjun Fan, Changzhen Zhang, Yu Qin, Xinhui Yin, Xinyi Dong, Nicolas Desneux, Hongxu Zhou

**Affiliations:** 1 Shandong Engineering Research Center for Environment-Friendly Agricultural Pest Management, China-Australia Cooperative Research Center for Crop Health and Biological Invasions, College of Plant Health & Medicine, Qingdao Agricultural University, Qingdao 266109, China; 2 Université Côte d’Azur, INRAE, CNRS, UMR ISA, 06000 Nice, France

**Keywords:** methyl eugenol, non-responsiveness, odorant binding protein, P450, trapping

## Abstract

**Simple Summary:**

*Bactrocera dorsalis* is a highly invasive polyphagous pest of fruit and vegetable, causing severe economic loss and trade restrictions by ovipositing inside hosts. Cryptic feeding habits of larval stages, strong flight ability of adults and prevalence of insecticide resistance reduce the efficiency of chemical control. Currently, the most cost-effective tool is olfaction-based trapping. Methyl eugenol (ME), a naturally occurring compound in some plants, alone or combination with insecticides has been widely used as a male attractant to monitor and control *B. dorsalis* populations for seven decades. Intense ME selection under laboratory conditions has resulted in the evolution of non- responsiveness in *B. dorsalis* and field management strategies based on ME has failed to eradicate recolonization of *B. dorsalis* in some islands. However, the levels of ME responsiveness in *B. dorsalis* field populations in China is unknown. In this study, we found that field populations had lower ME sensitivity compared to the susceptible strain. Furthermore, the results of olfactory organs, gene expressions and the bioassays showed that odorant binding protein (BdorOBP2, BdorOBP83b) and P450 may be involved in the lower sensitivity. The findings will guide the use of lures combined with insecticides and help to exploit molecular targets for the development of new attractants.

**Abstract:**

*Bactrocera dorsalis* is a notorious polyphagous pest in China, and its management strategies largely depend on methyl eugenol (ME), which has been widely used as an attractant to monitor and eradicate *B. dorsalis* populations for seven decades. However, the non-responsiveness levels in field *B. dorsalis* populations to ME is unknown. In this study, we monitored the response to ME in field populations from the four most heavily infested provinces in China, and the results showed that the populations had lower sensitivity to ME relative to GZS susceptible strain. The percent responsiveness of the lowest sensitivity population was 5.88-, 3.47-, and 1.47-fold lower relative to the susceptible strain at doses of 1, 10, and 100 µL of ME, respectively. Gene expression analysis and inhibitor assays further revealed that odorant binding protein (BdorOBP2, BdorOBP83b) and the P450 enzyme system may be associated with the lower response to ME. To our knowledge, this work is the first to report that the P450 enzyme system confers a lower responsiveness to lure insects. These findings provided valuable insights for exploiting ME non-responsiveness to protect sterile males from ME-based control strategies and the use of lures combined with insecticides.

## 1. Introduction

The oriental fruit fly, *Bactrocera dorsalis* (Hendel) (Diptera: Tephritidae) is an invasive pest that is widely distributed in tropical and subtropical regions [1]. Its females lay eggs directly into over 450 fruit and vegetable crops in which hatched larvae then feed and develop, causing heavy economic losses by making hosts inedible and increasing costs for export limitations due to quarantine measures [1,2]. Since its first record in early 1900s in Taiwan, *B. dorsalis* has been spreading: gradually into Hainan in the 1930s and then Guangdong, Guangxi, Yunnan, and Fujian in the 1950s and 1960s. In the 1980s and 1990s, the pest was widely distributed in the southern provinces in China [3,4,5]. Recently, owing to the climate change and transportation activities, the pest has expanded northward and is found in more than 65 countries on 6 continents, threating multiple agricultural and horticultural crops [6,7]. Due to the cryptic feeding habits of larval stages and pupating in the soil [8], management strategies mainly focus on the adults. Insecticides have been commonly used, but their extensive usage has led to the selection of resistant populations, reduced management efficacy [9], as well as multiple potential side effects of these compounds on non-target organisms [10,11,12]. Currently, olfaction-based adult trapping is the most cost-effective tool to control *B. dorsalis* [13]. 

*B.dorsalis* adult trapping largely relies on the use of methyl eugenol (ME), a naturally occurring compound found in more than 450 plant species [14]. ME has been widely and effectively used since the 1950s for the detection, monitoring and control of *B. dorsalis* mainly in three ways [15]. Firstly, it alone is used for early detection and monitoring of *B dorsalis* [16]. Secondly, it is combined with toxicants, such as malathion and spinosad (lure-and-kill) termed male annihilation technique (MAT) to manage *B dorsalis* [17,18]. The MAT was successfully used to eradicate outbreaks or isolated established populations of *B. dorsalis* from the Marianas Islands [17], the Amami Islands [19], the Okinawa Islands [20], the Lambay Island [21], and the Pacific Islands [22]. Thirdly, MAT is used to greatly reduce male abundance prior to the implementation of the sterile insect technique (SIT), which depends on an adequate ratio of sterile males to wild males [15]. Massive improvements in the combined control could be achieved by simultaneous application of MAT and SIT, but this requires weakening of the ME response in released sterile male flies [23].

Remarkably, *B. dorsalis* males are not only strongly attracted by ME but also voraciously feed on ME. After ingestion, ME in *B. dorsalis* is broken down into two main components, (E)-coniferyl alcohol (E-CF) and 2-allyl-4,-5-dimethoxyphenol (DMP), which are mediated by the cytochrome P450 enzyme system [24,25]. These components substantially enhance the mating performance of ME-fed *B. dorsalis* males compared with ME-deprived males [26]. Sterile males that previously fed on ME not only competed better with wild males for mating with wild fertile females but were also less responsive to the ME baits [27]. Such characteristics enable the possibility of using MAT and SIT simultaneously to effectively manage *B. dorsalis*, therefore some researchers put forward the strategy that sterile males are fed on ME before releasing to reduce or eliminate ME response [28,29]. 

It is known that ME has played a vital role in early detection and control of *B. dorsalis*, and the decrease of sensitivity to ME may dramatically affect the efficacy. However, it is not clear whether intense ME use in the fields for the last seven decades has caused the evolution of non-responsiveness to ME. Previous studies have shown that the responsiveness of male *B. dorsalis* to ME could be reduced via intense selection under laboratory conditions and MAT has failed to eradicate recolonization of *B. dorsalis* [30,31], suggesting that the characteristic of non-responsiveness to ME in individuals may have a heritable component. Therefore, in this study, we investigated the non-responsiveness levels of field *B. dorsalis* populations to ME, and further detected the expression levels of genes involved in detecting ME and the effect of P450 enzymes inhibitors on the response to determine the mechanisms of ME non-responsiveness. This information will contribute to understanding the evolution of non-responsiveness to ME and the management practices for this important agricultural pest.

## 2. Materials and Methods

### 2.1. Strains

One lab strain (GZS) and four field populations were used in the study. The susceptible GZS strain was originally collected from Guangzhou, China, in 2012 and reared in laboratories for >50 generations without exposure to insecticides and lures. Considering the invasive history of *B. dorsalis* (see introduction), the damage and ME use in orchard (personal communications), four field strains were collected from Timeng, Hainan province (TM population), Huizhou, Guangdong province (HZ population), Zhangzhou, Fujian province (ZZ population), and Nanning, Guangxi province (NN population) in China, between July and October 2020 from infested host fruits, including Guava (*Psidium guajava*) and papaya (*Carica papaya*). The infested fruits were incubated at 27 ± 1 °C, 75 ± 1% relative humidity, and a photoperiod cycle of 14 h L/10 h D before the pupae were collected. Emerging adults were maintained in cages (30 × 30 × 30 cm^3^) and supplied with a protein and sugar mixture (3 portions of glucose + 1 portion of peptone) and water for reproduction. After the adults were sexually mature, eggs were collected and hatched in bananas. Larvae were fed with artificial food (600 mL of water, 600 g of granulated sugar, 31 g of beer yeast powder, 0.5 g of sorbic acid, 0.6 g of methyl parahydroxybenzoats, 1 g of ascorbic acid, 235 g of wheat bran) in a plastic bucket (25 × 25 × 10 cm^3^).

### 2.2. Bioassays for ME Response

The bioassays of the ME response were conducted according to the methods of Liu et al. (2017) [31] with modifications. Twelve hours prior to an assay, 100 fifteen- to twenty-day-old mated males were transferred into a cage (30 × 30 × 30 cm^3^). The males were exposed to a trap containing a cotton wick with 1 mL of ME (>98% purity, Energy Chemical Company, Shanghai, China) dissolved in mineral oil (MO). Three doses of ME were used (1, 10 and 100 μL/mL), and a sample containing MO alone served as a control accordingly. The accumulated ME response was recorded at 6 h after trapping. The response to ME ratio was calculated using the following equation: percentage responsiveness = cumulative number of male flies trapped by ME/number of test male flies × 100. Three replicates were tested for each dose. The assays were run in a room with a temperature of 27 ± 1 °C with 75 ± 1% RH.

### 2.3. Assays for Olfactory Organ Detection of ME

A cage bioassay was used to assess the ability of males with the antennae removed, the proboscis removed, the maxillary palps removed, all three olfactory organs removed (antennae, proboscis and maxillary palps), or no organs removed to detect ME. In short, antennae, proboscises, maxillary palps, or all three olfactory organs were removed from fifteen and twenty-day-old males of the susceptible GZS strain by forceps under a stereomicroscope (Carl Zeiss, Jena Germany). Males with no organs removed were used as a control. All the males were then kept separately in cages with food and water for 12 h before being used for assays. For the assays, the males were offered a mixture of 100 μL ME and 900 μL MO, or 1 mL of MO alone (a control) dispensed on a cotton wick placed on a plastic cup trap (9 cm diameter). For each treatment, the total number of trapped males was recorded at 6 h. Twenty males were tested per treatment per replicate. Three replicates were tested for each treatment. During the experiments, the temperature and the relative humidity in the room were maintained at 27 ± 1 °C and 75 ± 1% RH, respectively. In all experiments, any of the removal of maxillary palps, proboscises and antennae was not found to affect the survival of the experimental flies compared to the intact males.

### 2.4. RNA Extraction and cDNA Synthesis

The assays of the olfactory organs showed that antennae, proboscises or maxillary palps played a vital role in ME detection in *B. dorsalis*. To determine whether different expression levels of olfactory genes contributed to ME non-responsiveness in field populations, three parts (antennae, proboscises and maxillary palps) of sexually mature males were prepared from the GZS strain and four field populations. The three dissected parts were frozen immediately in liquid nitrogen and stored at −80 °C until extraction. Three independent biological replicates were performed per strain or population. Total RNA was extracted using TRIzol reagent (Invitrogen, Carlsbad, CA, USA) following the manufacturer’s instructions. The purity of all RNA samples was assessed at an absorbance OD 260/280 ratio, while the integrity of the RNA was verified by 1% agarose gel electrophoresis. An additional gDNA eliminator was performed using RNase-Free DNaseI (Takara, Dalian, China). RNA was quantified by measuring the absorbance at 260 nm in a spectrophotometer (Thermo Nano Drop^TM^ 2000c; Santa Clara, CA, USA). cDNA was synthesized by using a PrimeScript^TM^ RT reagent Kit (Takara, Dalian, China) according to the manufacturer’s instructions.

### 2.5. Quantitative Real-Time PCR (qRT-PCR)

qRT-PCR was performed using a SYBR Premix ExTaq kit following the manufacturer’s instructions with a Stratagene Mx3000 P thermal cycler. The PCR master mix (20 μL) contained 10 μL of SYBR Green Supermix, 1 μL of cDNA templates, 1 μL of each forward and reverse primer (1 μmol/L), and 7 μL of double-distilled water. The following thermal program was executed: 95 °C for 15 min, followed by 40 cycles of 95 °C for 10 s, 55 °C for 20 s, 72 °C for 20 s, and a final melting cycle (from 55 °C to 95 °C). The α-tubulin gene of *B. dorsalis* was used as an internal control (GenBank accession number: XM_011212814). The relative gene expression levels were calculated by using the 2^−^^△△CT^ method of relative quantification. Samples for three biological and two technical replicates were used for each experiment. All primers that were used in this study are listed in Table 1.

### 2.6. Bioassay of P450 Inhibitors

**Figure 1:**
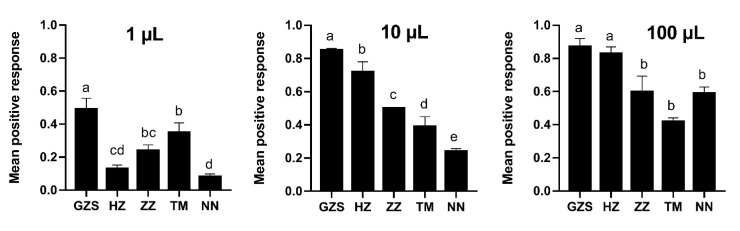
The response of *B. dorsalis* males from the GZS-susceptible strain and the four field populations to ME. The assay was conducted by using three doses of ME (1, 10, 100 µL) and MO alone served as controls accordingly in trap cages (30 × 30 × 30 cm^3^) for 6 h. No flies were trapped in the control MO cages. Data are presented as the mean ± SE. Different letters on the histogram bars indicate significant differences based on one-way ANOVA followed by Tukey’s HSD multiple comparison test (*p* < 0.05).

**Figure 2:**
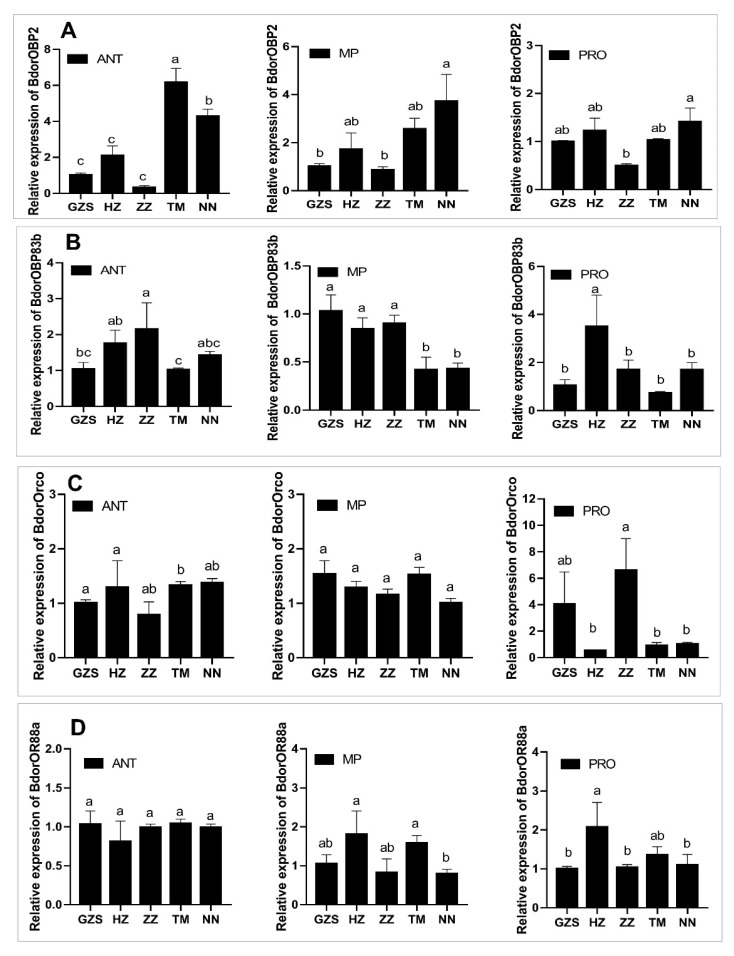
Expression profiles of four genes in the antennae (ANT), maxillary palps (MP) and proboscis (PRO) of *B. dorsalis* males from the GZS susceptible strain and the four field populations (**A**) BdorOBP2, (**B**) BdorOBP83b, (**C**) BdorOrco, and (**D**) BdorOR88a. Relative gene expression was measured by qRT-PCR, and values represent the means ± SEs for three independent replicates. Different letters on the histogram bars indicate significant differences based on one-way ANOVA followed by Tukey’s HSD multiple comparison test (*p* < 0.05).

It is well established that P450 enzymes are involved in pheromones and xenobiotic metabolism [32]. The strong attraction of *B. dorsalis* males to ME may be associated with their metabolites, which are more potent sex pheromone components mediated by P450 enzymes [25]. *B. dorsalis* has 101 P450 genes [33], and it is not clear that which P450 may be involved in ME metabolism. Therefore, we tested for the presence of P450-mediated resistance in the NN and TM populations that showed much higher ME resistant levels among field populations relative to the GZS strain and significantly different expression levels of OBPs based on the results (see Figure 1 and Figure 2), using assays with two types of P450 inhibitor piperonyl butoxide (PBO) and trichlorophenylpropynyl ether (TCPPE) [34,35]. PBO (95%) and TCPPE (95%) were purchased from Shanghai Aladdin Bio-chem Technology Co., Ltd. (Shanghai, China) and Beijing Jiuyan Biological Technology Co., Ltd. (Beijing, China), respectively. These bioassays were performed as described above, except that the inhibitor (PBO 2 μL/mL, TCPPE 4 μL/mL) was applied to each fly 2 h prior to the ME trap. For the inhibitor experiment, fifteen sexually mature adults were anesthetized on ice, and a 0.5 μL drop of inhibitor in acetone was applied to the thorax of 15- to 20-day-old males using a Hamilton PB-600 repeating dispenser equipped with a 25-μL syringe. For the ME response, the flies were treated with a mixture of 100 μL of ME and 900 μL of MO, or 1 mL of MO alone (a control) dispensed on a swab placed on a trap in the center of the cage, and the total number of trapped males was recorded at 6 h. Controls included an acetone application plus MO or inhibitor plus MO. Each treatment was replicated six times.

### 2.7. Statistical Analysis

One-way analysis of variation (ANOVA) followed by Tukey’s multiple comparison test were used to compare responsiveness to ME in the bioassays for ME response and the assays for olfactory organ detection of ME. An unpaired Student’s *t* test was used in the bioassay of P450 inhibitors. If data did not meet the normality or equality of the variance assumptions needed for a Student’s *t* test, the equivalent Mann-Whitney Rank Sum test was used instead. All statistical analyses were performed using the SPSS software v. 20. A *p* value < 0.05 was considered statistically significant.

## 3. Results

### 3.1. ME Response in B. dorsalis

To determine whether field *B. dorsalis* populations showed resistance to ME, three doses of 1, 10, and 100 µL of ME were assessed in four field populations and the lab strain within 6 h after treatment. The results showed that field *B. dorsalis* populations were resistant to ME, and overall, the NN population had the strongest resistance response to ME among the field populations relative to the GZS strain at this assay period and concentration range. For the 6 h trap, the percentage responsiveness in the lab GZS strain was 49% at concentrations as low as 1 µL of ME, which was significantly higher than those in field populations ranging from 8% (NN) to 35% (TM). Similar trends were found for both doses of 10 and 100 µL of ME, except in the HZ population. The percent responsiveness of the NN population was 5.88-, 3.47-, and 1.47-fold lower at doses of 1, 10, and 100 µL of ME relative to the GZS strain, respectively (Figure 1). In our assays, no flies were trapped in the control MO cages.

### 3.2. Olfactory Organs Detecting ME in B. dorsalis

**Figure 3:**
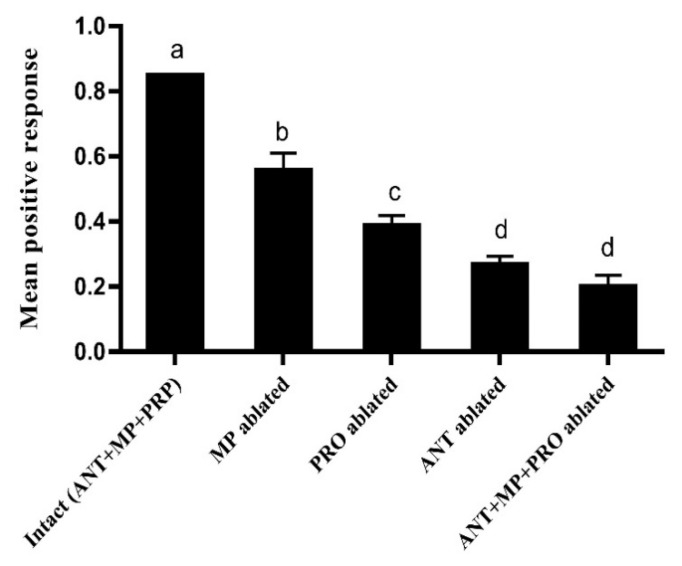
The effect of olfactory organs on the response of *B. dorsalis* males to ME. Assays were performed in trap cages (30 × 30 ×30 cm^3^) after the removal of the antennae (ANT), maxillary palps (MP) and proboscis (PRO) in a mixture of 100 μL ME and 900 μL MO, or 1 mL of MO alone (a control). No flies were trapped in the control MO cages. Data are presented as the mean ± SE. Different letters on the histogram bars indicate significant differences based on one-way ANOVA followed by Tukey’s HSD multiple comparison test (*p* < 0.05).

Our results showed that removal of any or all olfactory organs (antennae, proboscises and maxillary palps) had a significant effect on male response to ME over 6 h of exposure. The intact, untreated males (85%) were the most attracted to ME, followed by the males with maxillary palps removed (56%), males with the proboscis removed (39%), males with antennae removed (27%), and males with all three organs removed (20%) in the 6 h trap. The effect ratios of the removal of antennae had no significant difference compared with the removal of all three organs (Figure 3). Altogether, these results suggested that antennae, proboscises and maxillary palps are involved in the detection and response to ME and other organs in mature males of *B. dorsalis* may also have a role in detecting ME.

### 3.3. Expression Patterns of Genes Involved in ME Detection in B. dorsalis

The expression patterns of four genes showed that the common trends were that OBPs had significant differences in field populations relative to the GZS strain in antennae, maxillary palps and proboscises. For BdorOBP2, higher expression levels were found in the antennae of the NN and TM populations, and they were 4.27- and 6.16-fold higher than those of the GZS strain, respectively; its expression levels were not significantly different in proboscises and maxillary palps between the GZS strain and the field populations except for in the NN population (Figure 2A). For BdorOBP83b, the expression levels in the maxillary palps were lower in the NN and TM populations than in the GZS strain, while the ZZ population and the HZ population had significantly higher expression in the antennae and the proboscises, respectively, than the GZS strain (Figure 2B). For BdorOrco and BdorOR88a, no significant difference in the antennae, proboscises or maxillary palps was found between field populations and the GZS strain except for BdorOR88a in proboscis of the HZ population (Figure 2C,D).

### 3.4. P450-Mediated Resistance Response to ME in B. dorsalis

**Figure 4:**
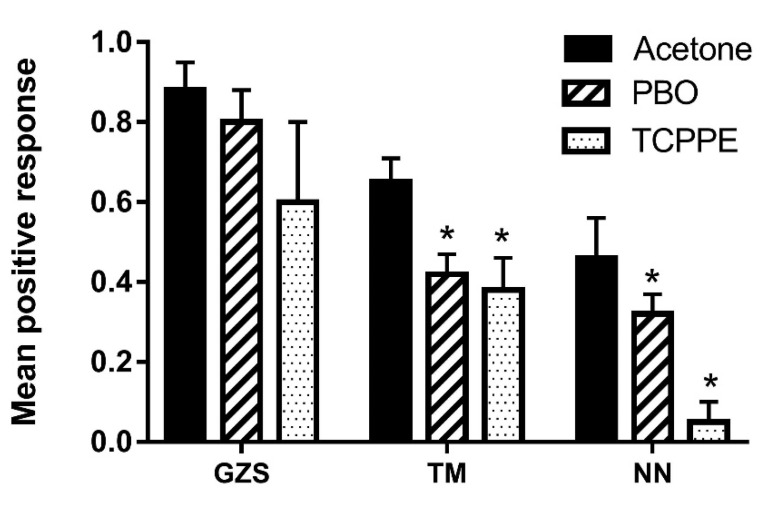
The effect of P450 inhibitors on the response of *B. dorsalis* males from the GZS susceptible strain and the two field populations to ME. Assays were performed in trap cages (30 × 30 × 30 cm^3^) using a mixture of 100 μL ME and 900 μL MO, or 1 mL of MO alone (a control). No flies were trapped in the control MO cages. Data are presented as the mean ± SE. The asterisks on the histogram bars indicate significant differences compared with the control (acetone) within each *B. dorsalis* line according to Student’s *t* test (*p* < 0.05).

To determine whether P450 is mediated by a lower sensitivity to ME in *B. dorsalis* field populations, we examined the effect of the P450 inhibitors PBO and TCPPE on the response to ME in the GZS-susceptible strain and the two field populations that showed a lower sensitivity response to ME (Figure 1). For the GZS strain, there was no significant difference in response to ME between the control (acetone) group and the treated groups (PBO or TCPPE). In contrast, for the two field populations, both PBO and TCPPE significantly decreased the response to ME relative to the control group, and a higher difference ratio was observed in the TCPPE-treated group in the NN population relative to the TM population (Figure 4).

## 4. Discussion

Methyl eugenol (ME), a plant secondary metabolite used as a lure, has been widely used for the detection, monitoring and management of *B. dorsalis*. ME used in combination with toxicants termed the male annihilation technique (MAT) has been used successfully to eradicate established populations of *B. dorsalis* in some areas but has failed to eradicate recolonization by *B. dorsalis* [15]. Here, we investigated the responsiveness of four field populations of *B. dorsalis* to ME collected in 2020 in China and revealed that the field populations had lower responsiveness to ME than the lab strain (Figure 1). These results suggest that the intense use of ME may have caused non-responsiveness to evolve in field populations. These results are similar to reports in which the responsiveness of *B. dorsalis* to ME could be reduced via artificial selection (5–12 generations) under laboratory conditions [30,31]. The findings further support that it is inefficient to only depend on MAT to control *B. dorsalis* populations. Moreover, Shelly (1997) [30] and Liu et al. (2017) [31] found that the proportion of non-responders remained stable (not continually decreasing). Whether the proportion of non-responsiveness of field populations to ME can increase after ME selection requires further study.

Olfactory perception in insects is first recognized by their peripheral olfactory organs, mainly including the antennae, maxillary palps and proboscis, which have olfactory receptors [36]. ME detection has focused largely on the role of the antennae, followed by the maxillary palp [37], and the involvement of the proboscis is poorly understood. In cage bioassays (<30 cm), our results showed that in addition to the antennae and the maxillary palp, the proboscis was involved in detecting and responding to ME, in which the antennae had the greatest ability, followed by the proboscis and the maxillary palps, suggesting that the proboscis is also an important part of the circuitry involved in ME detection by *B. dorsalis* males. Furthermore, antenna removal and removal of all three organs had the same effect on the ME response, indicating antenna plays the most important role in detecting ME in *B. dorsalis*. Males with all three organs removed were still attracted to the ME, while none of the males with intact antennae responded to the control of MO (Figure 3), suggesting that sensilla involved in the response to the ME may be also present in other organs. Our results have both similarities and differences to a previous report by Chieng et al (2018) [37] (only antennae and maxillary palps were studied), in which antennae had a larger role than maxillary palps in response to ME, and none of the male *B. dorsalis* with both antennae and maxillary palps removed responded to ME at short distances (<40 cm). The different results may be due to a longer observed time in our study (6 h) relative to their findings (20 min). Interestingly, it has been reported that male *B. tryoni* primarily use their maxillary palps and not their antennae to detect and respond to raspberry ketone (RK: a phenylbutanoid), another known male-specific attractant in tephritids [38]. The divergent behavioral phenotypes may be due to different olfaction detection, which has differences in the genes expressed in the olfactory organs of different species.

Currently, four chemosensory proteins have been reported to be involved in the detection of ME, including odorant binding protein BdorOBP2 [39], BdorOBP83b [40], odorant receptor BdorOR88a [41], and odorant receptor co-receptor BdorOrco [42]. Among them, the lower expression levels of BdorOBP2 were found in ME-non-responsive male antennae than ME-responsive males after ME selection under laboratory conditions for five generation selections [31]. Furthermore, BdorOBP2 gene silencing by RNAi or knockout by CRISPR/Cas9 significantly reduced the response of *B. dorsalis* to ME [31,39]. However, in our study, BdorOBP2 had higher expression levels in antennae and maxillary palps of males which were lower sensitivity to ME (Figure 2A). The inconsistence likely results from the possibility that other mechanisms are contributed to non-responsive to ME or that BdorOBP2 is also involved in other phenotypes (e.g. insecticide resistance) in field populations based on previous report in which DcitOBP2 is potentially involved in reduced insecticide susceptibility as a buffering protein in *Diaphorina citri* [43]. The hypothesis requires further study. Moreover, BdorOBP83b had the lower expression levels in maxillary palp of lower sensitivity of males (Figure 2B). The similar correlation was found in previous report in which the reduction in BdorOBP83b transcript abundance by RNAi led to a decrease in responses to ME in *B. dorsalis* [40]. Previous reports show that ME may induce the expressions of BdorOR88a and BdorOrco in antennae of *B. dorsalis* males and reducing their transcript levels led to a significant decrease in the males’ responsiveness to ME [41,42]. Our results showed their expression had no significant difference in the tested populations, suggesting the odorant receptor and its co-receptor may do not contribute to the lower ME responsiveness in field populations in expression levels. Similar results were found in target-site resistance of insecticide resistance mechanisms [44].

P450 enzymes are involved in pheromone biosynthesis and transportation [45,46]. Previous studies have shown that the strong attraction of *B. dorsalis* males to ME may be due to its metabolites (E)-coniferyl alcohol (E-CF) and 2-allyl-4, -5-dimethoxyphenol (DMP) mediated by the P450 enzyme system after ingestion [24,25]. Specifically, upon consumption, ME is quickly bio-transformed to E-CF and DMP in crops, then transported to rectal glands through hemolymphs not midguts, and subsequently release during courtship at dusk to function as male aggregation (DMP) and sex pheromones (E-CF) to enhance male mating competitiveness [26,47]. Our results showed that PBO and TCPPE significantly increased ME-non-responsiveness in the NN and TM populations. In contrast, PBO and TCPPE did not affect responsiveness to ME in the GZS strain (Figure 4). These results suggested that the P450 enzyme system may be involved in ME non-responsiveness in field *B. dorsalis* populations, but the mechanisms by which P450 enzymes regulate ME metabolism in the populations require further study. The metabolic pathways of ME in *B. dorsalis* suggest P450 enzymes in olfactory organs (quickly detected metabolites in crops) are more likely to be associated with the lower ME sensitivity, and OBPs in the olfactory organs are responsible for the first step of odorant perception detection [48]. Thus, further studies require to investigate if the interactions of OBPs and P450 in the olfactory organs regulate the ME response. Moreover, *B.* *dorsalis* males are strongly attracted by ME, pre-feeding on ME will decrease the response to it in laboratory bioassays and in field trapping studies [27]. Thus, we wonder whether ME-non-responsiveness mediated by the P450 enzyme could be combined with sterile technology for the area-wide management of *B. dorsalis* by decreasing the response to ME in sterile males. Furthermore, because ME in mammals has a specific 1′-hydroxy metabolite potentially responsible for hepatoxicity and carcinogenicity, it has been set as a 2B carcinogen by the International Agency for Research on Cancer [49,50]. The findings may aid to explore analogs and derivatives to overcome the limitations.

## 5. Conclusions

In conclusion, we report that the responsiveness to ME in *B. dorsalis* field populations collected in China has a lower ME sensitivity relative to a susceptible strain. Cage assays show that all three olfactory organs, antennae, proboscis and maxillary palps, are involved in detecting and responding to ME over short distances, and the proboscis has a greater role than the maxillary palps. Gene expression analysis and inhibitor assays further reveal that OBPs (BdorOBP2, BdorOBP83b) and the P450 enzyme system may be associated with a lower response to ME in field populations. Future studies are required to determine which P450 genes confer less non-responsiveness. Altogether, our results aid in the understanding of management practices of *B. dorsalis*.

## Figures and Tables

**Table 1:** Primers used for gene expression detection by qRT-PCR.

Gene Name	NCBI Accession Number	Nucleotide Sequences (Forward)	Nucleotide Sequences (Reverse)
OBP2	KC559113	GTTTTGCTAGCCTTTGTCGC	CTTGCATGCACTTGGAGAAG
Orco	MT474521	CCTATTCGTGCCACTGGTATGAT	AGAACCGATGCAAACAAGTCC
OR88a	KP743732	TGTATGCTTCGTGGTTACCG	CATCCGGCACATTCATTTCC
OBP83b	KP743700	CTCCCGAAAGACTCTCCTGG	GAACATCCCCATCGCTGAAC
α-tubulin	XM_011212814	CGCATTCATGGTTGATAACG	GGGCACCAAGTTAGTCTGGA

## Data Availability

The data presented in this study are available on request from the corresponding author.
